# Effect of Surfactants on the Molecular Structure of the Buried Oil/Water Interface

**DOI:** 10.1002/anie.202110091

**Published:** 2021-09-14

**Authors:** Saman Hosseinpour, Vanessa Götz, Wolfgang Peukert

**Affiliations:** ^1^ Institute of Particle Technology (LFG) Interdisciplinary Center for Functional Particle Systems (FPS) Friedrich-Alexander-Universität Erlangen-Nürnberg (FAU) Cauerstrasse 4 91058 Erlangen Germany

**Keywords:** emulsions, sum frequency generation (SFG) spectroscopy, surface analysis, surface tension, surfactants

## Abstract

The oil/water interface, for instance in emulsions, is often stabilized by surfactants. Hence, the co‐existence of oil, water, and surfactant molecules at the buried oil/water interface determines macroscopic properties such as surface tension or emulsion stability. Utilizing an inherently surface sensitive spectroscopic method, sum frequency generation (SFG) spectroscopy, we show that adsorption of an anionic surfactant to the buried oil/water interface increases the magnitude of the interfacial electric field. Meanwhile, the degree of ordering of the interfacial oil molecules increases with the surfactant concentration owing to the intercalation of aliphatic chains of interfacial oil and surfactant molecules. At sufficiently high surfactant concentrations, the interfacial charge reaches a maximum value and the interfacial oil molecules arrange in a fully ordered conformation, a state which coincides with the significant decrease in interfacial tension and increased emulsion stability.

## Graphical abstract

The increased ordering of oil molecules' aliphatic chains and the enhanced alignment of water molecules at the oil/water interface was revealed upon increasing adsorption of anionic surfactant using sum frequency generation (SFG) spectroscopy. The surfactant coverage inducing the highest‐ordered oil molecule configuration at the interface matches a significant reduction of interfacial tension and high emulsion stability.

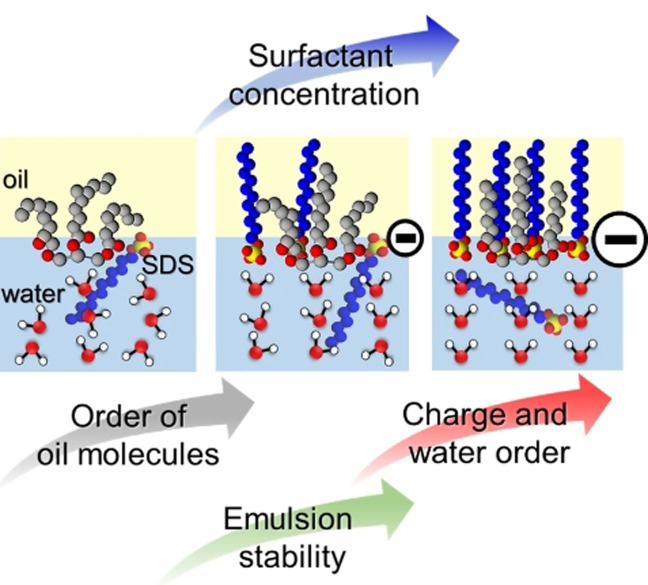

## Introduction

Oil/water interfaces are omnipresent in our life, e.g., at the surface of cell membranes, in dairy and other food products, as well as in technological fields such as oil refinery and pharmaceutics. Often, the boundary between water and oil, for instance in an emulsion, is stabilized by natural or synthetic amphiphilic species like proteins, alcohols, polymers, or surfactants. The stability and other physicochemical properties of emulsions are thus controlled by the chemical composition, molecular arrangement, and electrostatic interactions at the oil/water interface. For example, surfactants tend to decrease the interfacial tension between oil and water and hinder the coalescence of the dispersed phase in an oil‐in‐water (O/W) or water‐in‐oil (W/O) emulsion.^[1]^ Despite many studies that have been performed to evaluate the effect of surfactants on the short‐ and long‐term stability of emulsion systems, the detailed molecular level information regarding interactions between different species and their role on the stability of emulsion systems is scarce.

Sum frequency generation (SFG) spectroscopy is an ideal tool for studying the molecular interactions between different species at any surface and interface accessible by laser light, including buried oil/water interfaces. The inherent surface sensitivity and surface specificity of SFG allow for the characterization of molecules residing at the boundary between two phases without the contribution of the bulk species to the SFG signal. SFG spectra not only provide information on the type of the species residing at surfaces and interfaces, but they also contain information on the local environment, molecular orientation, and degree of ordering of interfacial molecules.^[2]^ For instance, the structure and orientation of alkanes with different chain lengths,^[3]^ the interactions of oppositely charged surfactants,^[4]^ as well as the temporal evolution of their structure^[5]^ at the solid/liquid or liquid/gas interfaces have been investigated by SFG spectroscopy. Miranda et al. studied the conformation, degree of order, and orientation distribution of surfactant monolayers in contact with long and short alkanes, CCl_4_, water, and alcohol.[^6^, ^7^] Messmer et al.^[8]^ pioneered the SFG studies of surfactants at the interface between two immiscible liquid phases and observed a relatively disordered structure of sodium dodecyl sulfate (SDS) at the water/CCl_4_ compared to that adsorbed at the water/air or solid/water interface. The same research group studied the co‐adsorption of a surfactant and alcohol as well as the ordered polyelectrolyte assembly at the oil/water interface.^[9]^

As evidenced by these studies, the molecular information that is obtained regarding the adsorption conformation of surfactants at an interface (e.g., solid/liquid or liquid/gas) is not directly transferrable to another interface (e.g., oil/water in an emulsion system). Moreover, SFG investigations at the oil/water interface are often limited to model hydrophobic phases such as CCl_4_ or alkanes, which are not suitable for product formulation in food, cosmetics, or pharmacy due to health and environmental considerations. Finally, in most of the previously mentioned studies, the focus has been to determine the conformation and molecular organization of the surfactant molecules at the oil/water interface. Nevertheless, the adsorption of surfactants at this interface also imposes changes in the structure and conformation of oil molecules that reside at the oil/water interface. As revealed by Roke's group, using sum frequency scattering (SFS), at the interface of surfactant stabilized single‐chain alkane droplets in water (nanodroplets of ≈100 nm in diameter) very few chain defects exist in the oil phase, as opposed to the surfactant chain conformation.^[10]^ The molecular interactions between oil and surfactant for other emulsion systems (e.g., with different oil structures or droplet sizes) lead to significant changes in elasticity of the interfacial layer and consequently affect the macroscopic emulsion stability.^[11]^

In the present study, we utilize complementary SFG and interfacial tension (IFT) measurements to unravel the electrostatic and molecular interactions between medium‐chain triglyceride (MCT) oil molecules and an anionic surfactant, SDS, at the buried oil/water interface. In contrast to the previously investigated model oil systems, MCT is widely applicable in the formulation of emulsion systems in the cosmetics and food industries. Hence, the impact of surfactant concentration on the molecular arrangement and conformation of the interfacial MCT molecules will be the focus of this study. These molecular interactions at the buried oil/water interface well describe the macroscopic stability of emulsions (with the droplet diameters of >1 μm). As the molecular interactions between oil, surfactant, and water molecules determine the final stability of the emulsion systems, the information obtained in this fundamental study paves the way for the systematic design of emulsion systems with appropriate short‐ and long‐term stability.

## Results and Discussion

**Figure 1:**
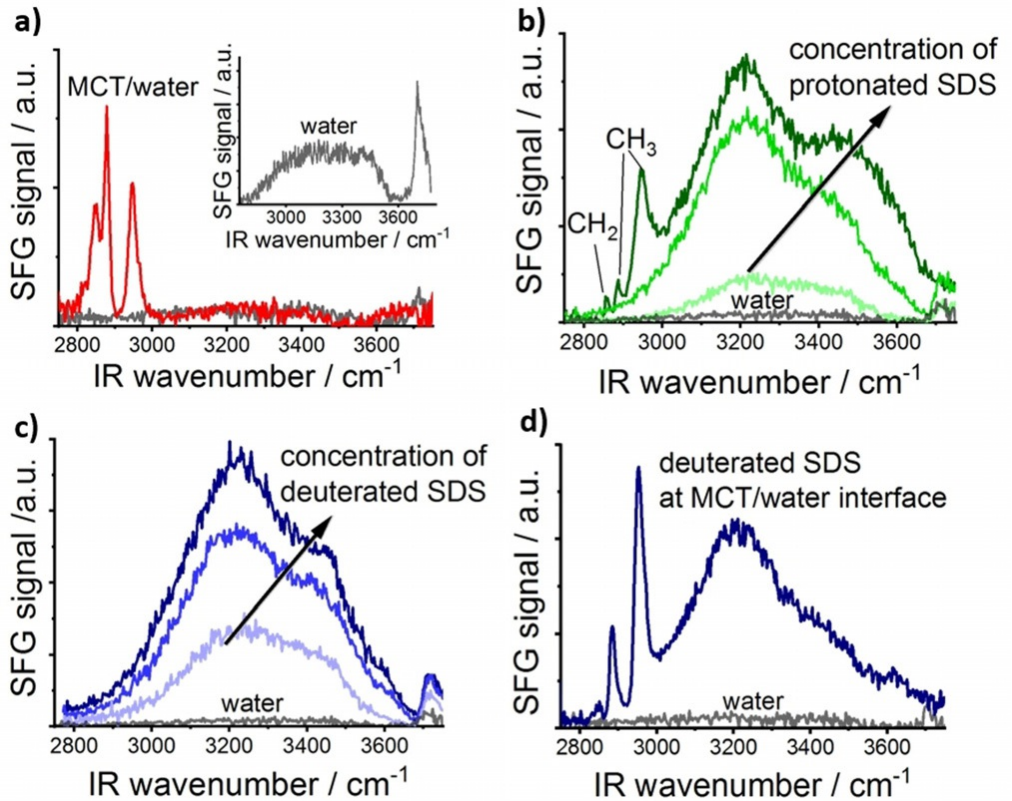
a) SFG spectrum of the buried MCT/water interface. The inset shows the SFG spectrum at the neat water/air interface. b) and c) SFG spectra of protonated and deuterated SDS (32 to 191 μmol L^−1^) at the water/air interface, respectively. d) SFG spectrum of MCT oil and deuterated SDS (dSDS, 32 μmol L^−1^) at the buried oil/water interface. In all panels, the SFG spectrum of the neat water/air interface is provided as a reference.

In order to evaluate the individual characteristics and structure of MCT oil and SDS, SFG measurements were performed on MCT, SDS, and its fully deuterated counterpart (dSDS) at the water/air interface and the corresponding spectra in the CH−OH stretching region (i.e., from 2750 to 3750 cm^−1^) are presented in Figure 1 a–c. The inset in Figure 1 a represents the SFG spectrum of neat water/air interface. In this spectrum, the sharp peak at ≈3700 cm^−1^ originates from the vibration of the non‐hydrogen bonded dangling OH protruding toward air. This peak, also known as the free OH peak, is characteristic of neat water surface and disappears in the presence of surface adsorbates such as surfactants or oil molecules at the water surface. The broad band in the SFG spectrum of the neat water/air interface (3100–3500 cm^−1^, henceforth referred to as OH‐signal) originates from hydrogen‐bonded interfacial water molecules and is often described with the contribution of strongly and weakly hydrogen‐bonded water molecules^[12]^ (also referred to as “ice‐like” and “liquid‐like” water, respectively). The exact position of these peaks depends upon the strengths of hydrogen bonding of the interfacial water molecules, whereas the intensity of the SFG signal from interfacial water molecules (i.e., OH‐signal) highly depends on the magnitude of the electric field at the surface, which can align the polar water molecules with H‐up or H‐down configuration in negatively and positively charged surfaces,^[13]^ respectively. Hence, the SFG spectrum of the neat water/air interface is included in all spectra as a reference. It has been shown earlier that ions, ionic surfactants, or charged proteins can electrify the interface and enhance the intensity of the OH‐signal in SFG spectra due to an increased number of aligned water molecules.^[14]^ Accordingly, SFG spectroscopy has been utilized to investigate the acid–base equilibrium of a cationic surfactant^[15]^ or to determine the isoelectric point of protein solutions at the water/air interface^[16]^ and at hydrophobic interfaces.^[17]^

The red (bright) curve in Figure 1 a displays the SFG spectrum of a thin MCT oil layer deposited on top of the water phase. Three peaks in the CH stretch region (i.e., 2800–3000 cm^−1^) dominate the spectrum; the symmetric methylene vibration, CH_2,sym_ at ≈2860 cm^−1^, the symmetric methyl vibration, CH_3,sym_ at ≈2884 cm^−1^, and the Fermi resonance signal of the CH_3_ group, CH_3,FR_ at ≈2946 cm^−1^.^[18]^ As shown in this Figure, the intensity of the OH‐signal from the MCT/water interface is comparable to that of the neat water/air interface, reflecting the uncharged nature of MCT oil.

In contrast to the uncharged MCT, SDS molecules contain an anionic head group, adsorption of which to the water surface increases the electric field at the water/air interface and strongly aligns the water molecules with their oxygen away from the surface.^[19]^ This preferred alignment of interfacial water molecules increases the OH‐signal intensity compared to that for the neat water/air interface, as depicted in Figure 1 b. With increasing the concentration of SDS the magnitude of the electric field at the water/air interface and consequently the number of aligned water molecules increase, leading to further enhancement of the OH‐signal intensity (see Equation (2) in the SI). At low SDS concentrations, the conformational order in the hydrophobic tails of interfacial SDS molecules is negligible and SDS molecules are randomly arranged in plane. Once the bulk concentration of SDS reaches a threshold value, the van der Waals (vdW) force between the hydrophobic tails of the interfacial SDS molecules increases, leading to a relative ordering of SDS molecules at the solution/air interface. Thereby, methyl and methylene vibrations appear in the SFG spectra consistent with previous studies.^[20]^ The presence of the methylene signal (at ≈2860 cm^−1^) denotes the presence of *gauche* defects in the adsorbed SDS layer at the solution/air interface.

As is shown in Figure 1 a,b, both MCT oil and SDS can generate SFG signals in the CH‐region. Therefore, it is difficult to discern their individual contributions to the SFG spectra of systems including their mixture. In molecular spectroscopy of organic molecules, however, isotopic labeling is a common method to separate the corresponding signals between labeled moieties and their protonated counterparts. For instance, replacing hydrogen (H) atoms in a hydrocarbon chain with deuterium (D) atoms shifts the molecular frequency of the labeled species to lower wavenumbers.[^7^, ^21^] The SFG spectra of dSDS in normal and heavy water (H_2_O and D_2_O) have been reported in both CH−OH (i.e., 2800–3700 cm^−1^) and CD−OD (i.e., 2000–2800 cm^−1^) spectral regions.^[18]^ Hence, we utilized dSDS for SFG measurements involving oil, water, and surfactant. In Figure 1 c, the SFG spectra of dSDS with different bulk concentrations are provided in the CH−OH stretching region. Since dSDS contains the same ionic head group as normal SDS (i.e., SO_4_^−^), its adsorption to the water/air interface electrifies the surface in the same manner as was explained for SDS (Figure 1 b), that is, the OH‐signal intensity increases with increasing the bulk concentration of dSDS.

In Figure 1 d, a representative SFG spectrum of the MCT oil/water interface in the presence of dSDS is provided. In this spectrum, the signal in the CH‐region is solely generated from the interfacial MCT oil molecules, whereas the signal in the OH‐region is related to the surface‐adsorbed dSDS molecules and can be used as a measure of the magnitude of the electric field at the interface. Hence, we can identify the individual contribution of surface species (i.e., MCT oil, water, and surfactant) at the buried interface between oil and water.

**Figure 2:**
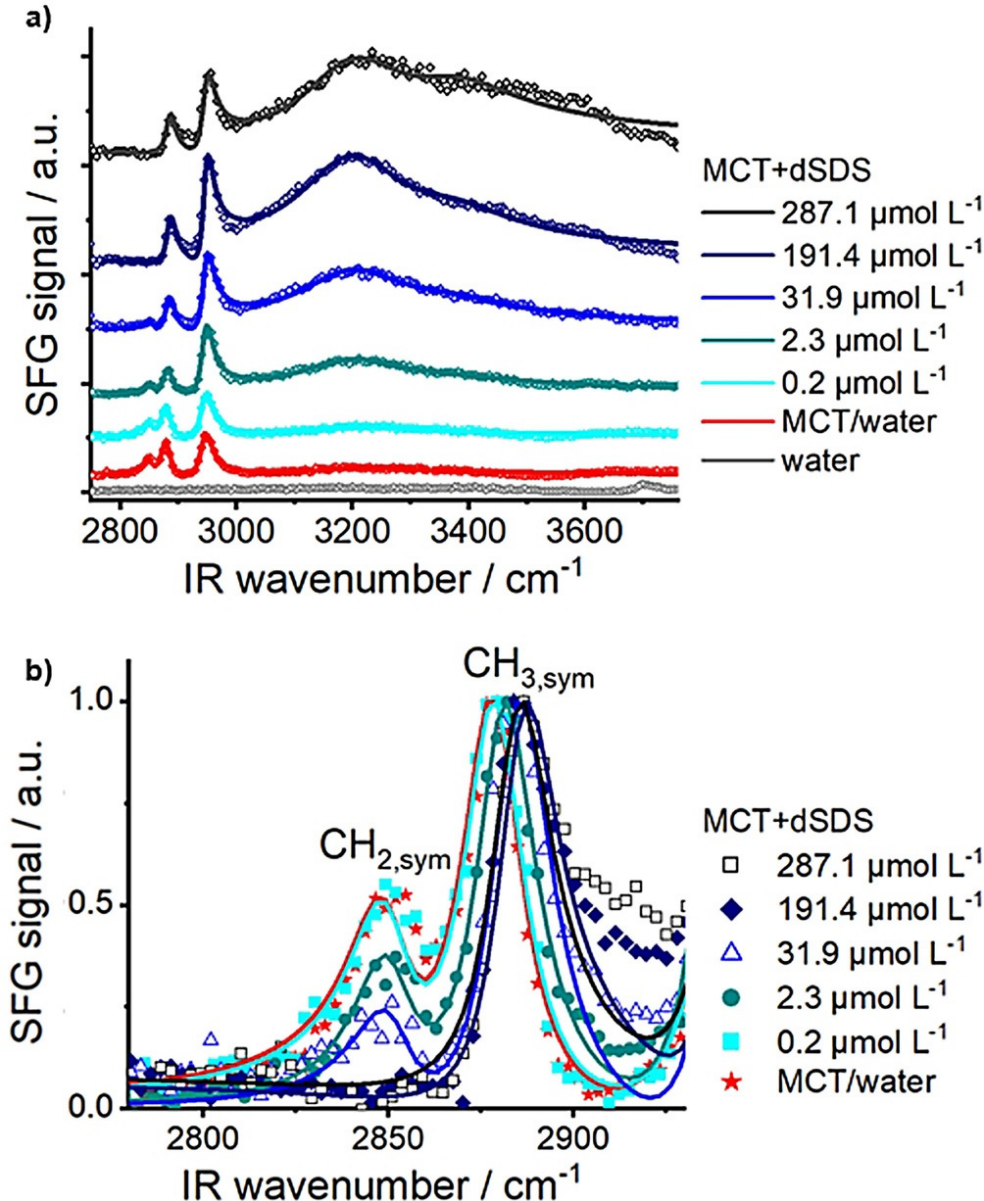
Measured SFG spectra (symbols) and fitted spectra (lines). a) Effect of bulk dSDS concentration on the SFG spectra of the buried MCT oil/water interface. SFG spectra are stacked vertically for clarity. SFG spectrum of water is presented as a reference. b) Normalized SFG spectra in the CH‐region.

We performed SFG measurements at the buried oil/water interface with different concentrations of surfactant in the bulk solution to determine the impact of the surfactant on the structure of MCT oil. Figure 2 a shows the evolution of SFG spectra of MCT oil/water interface with increasing bulk concentration of dSDS. The presented spectra are recorded at equilibrium and the SFG spectrum of the neat water/air interface is provided at the bottom of this Figure for comparison. As can be seen from these spectra, up to a bulk dSDS concentration of 0.2 μmol L^−1^, the SFG spectrum of the MCT‐water‐surfactant system and that of the MCT–water system without surfactant are almost identical. With increasing bulk concentration of dSDS and surpassing 2.3 μmol L^−1^, the OH‐signal intensity starts to increase. The maximum intensity of the OH‐signal is obtained at the bulk dSDS concentration of ≈190–280 μmol L^−1^. As was explained earlier, adsorption of dSDS to the oil/water interface and the buildup of an electrified interface are responsible for the increased alignment of water molecules, which results in the enhancement of SFG OH‐signal intensity.

The observed increase in the OH‐signal intensity upon increasing dSDS bulk concentration is accompanied by noticeable changes in the SFG signal in the CH‐region. For better visualization, the SFG spectra in the CH‐region are normalized to the maximum peak intensity (i.e., CH_3,sym_ peak) and are provided in Figure 2 b. From this Figure, it is clear that the ratio between the peaks corresponding to CH_2,sym_ and CH_3,sym_ stretching vibrations decreases dramatically as the bulk dSDS concentration increases.

The commencement of the change in the CH_2,sym/_CH_3,sym_ peak ratio of MCT oil (i.e., dSDS concentration ≈2 μmol L^−1^) coincides with the onset of the increase in the OH‐signal intensity. At the highest dSDS concentrations (i.e., ≳190 μmol L^−1^), the peak corresponding to the CH_2,sym_ vibration from MCT oil completely vanishes, suggesting the change in the conformation of interfacial oil molecules, as will be discussed in the following.

In the *all trans* conformation, e.g., in methylene‐terminated aliphatic self‐assembled monolayers or surfactants, in which the hydrocarbon chains are highly ordered, the CH_2_‐groups are symmetrically arranged in pairs along the chain.^[22]^ Due to this local centrosymmetric environment, the CH_2_ vibrations become SFG inactive and only the terminal CH_3_‐groups of the alkyl chain contribute to the SFG spectra. In contrast, at a low degree of ordering, i.e., alkyl chains that have *gauche* defects and kinks in their structure, the CH_2_‐vibrations are also SFG active and contribute to the SFG signal together with the CH_3_‐group.^[23]^ Therefore, the degree of ordering and the extent of *gauche* defects in an alkyl chain have been estimated using the intensity ratio of the CH_2,sym_ and CH_3,sym_ peaks in the SFG spectra for surfactants at the liquid/air interface^[24]^ or at solid/hydrophilic surfaces.^[25]^ Here, utilizing the relative intensities of CH_2,sym_ and CH_3,sym_ we evaluated the influence of the surfactant concentration on the conformation and degree of ordering in the hydrocarbon chains of the MCT oil.

**Figure 3:**
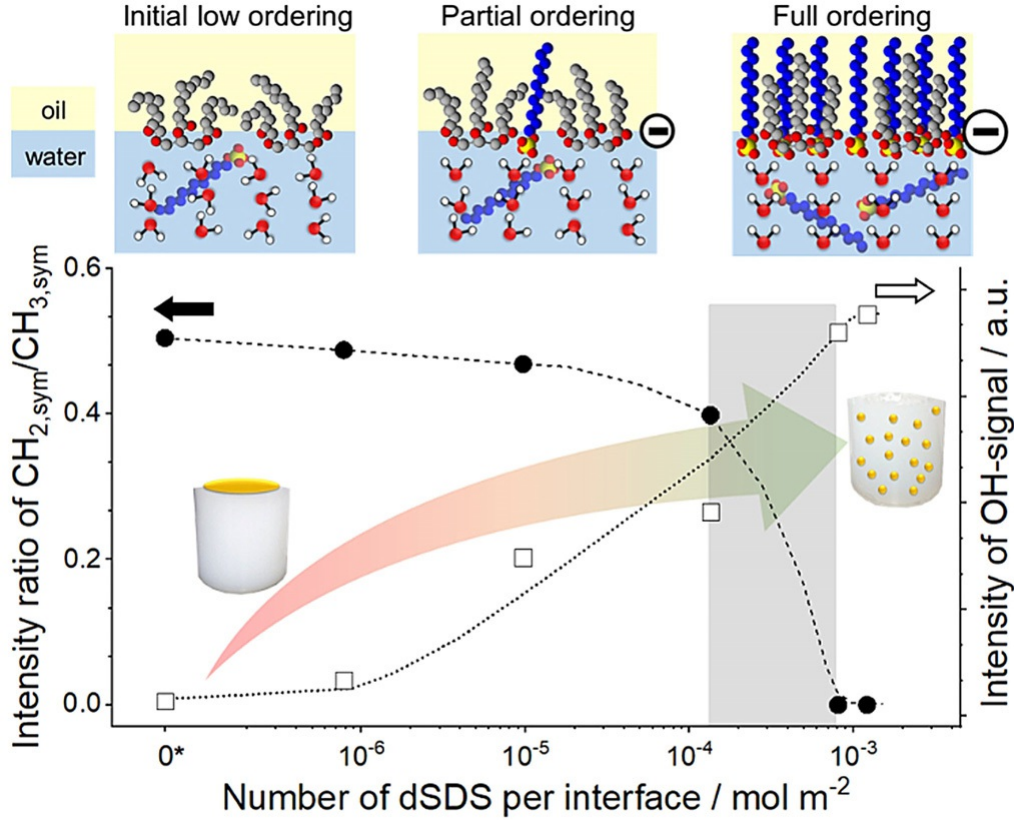
Evolution of CH_2_/CH_3_ peak intensity ratio (full symbols, left axis) of MCT molecules at the buried oil/water interface and the corresponding OH‐signal intensities (open symbols, right axis) as a function of dSDS coverage. Lines are to guide the eyes. The corresponding values in the absence of surfactant are displayed as 0* in the logarithmic axis. The arrangement of interfacial molecules is schematically depicted on top. The arrow depicts the increased emulsion stability with increasing surfactant coverage. The right scale does not start from 0.

To accurately analyze the change in intensity of the individual peaks, we fitted the SFG spectra using the procedure described elsewhere^[26]^ and provided the results in Figure 3 (details of peak assignments, and fitting parameters are given in the SI). As seen in this Figure, the CH_2,sym_/CH_3,sym_ peak ratio of MCT oil is decreasing with increasing the dSDS coverage. MCT oil deposited on the neat water surface (in the absence of surfactant in solution) exhibits the highest CH_2,sym_/CH_3,sym_ ratio, thus the lowest degree of ordering and the largest number of *gauche* defects along its alkyl chains. The CH_2,sym_/CH_3,sym_ peak ratio changes insignificantly with the addition of 0.2 μmol L^−1^ dSDS (7.8×10^−7^ mol m^−2^) in the bulk water phase. With further increasing the surfactant concentration, however, the CH_2,sym_/CH_3,sym_ ratio decreases significantly due to the increased order of the hydrocarbon chains in the interfacial MCT oil molecules and removal of *gauche* defects in their hydrocarbon chains. At bulk dSDS concentration of ≳190 μmol L^−1^ (8.1×10^−4^ mol m^−2^), the CH_2,sym_/CH_3,sym_ peak ratio is negligible and the interfacial MCT oil molecules adopt an *all trans* conformation, hereafter referred to as “full ordering”.

The observed increased degree of ordering in the interfacial oil molecules with increasing the surfactant concentration can be explained by the intercalation of aliphatic chains of surfactant molecules between the chains of interfacial MCT oil molecules (see Figure 3), which decreases the available area per molecule at the buried oil/water interface and increases the vdW interactions. This conclusion is consistent with quantum chemical calculations and thermodynamic evaluations on long alkane chains of mixed monolayers of surfactants, confirming cooperative interactions between surfactant and alkane molecules at the buried oil/water interface.^[27]^ Other studies also indicate that the presence of surfactant molecules at the oil/water interface alters the organization of oil molecules at the buried oil/water interface. For instance, recent SFG experiments^[28]^ and molecular dynamic studies^[29]^ revealed that in the absence of surfactants, oil molecules lie rather parallel with respect to the oil/water interface, whereas surfactant adsorption to this interface changes the alignment of the oil molecules to a more perpendicular orientation.

The high surfactant concentrations in this Figure (i.e., ≈190 and ≈287 μmol L^−1^) are far below the critical micelle concentration (CMC) value of SDS (i.e. ≈2–4 % of CMC) but contain enough SDS molecules to fully cover the small surface area of the flat oil/water interface, assuming that all molecules go to the surface and the space needed for one SDS molecule is 18.4 Å^2^.^[30]^ Nevertheless, it has already been shown that only a fraction (approximately 40 %) of SDS molecules go to the water/hexadecane interface of an emulsion.^[31]^ Other studies have also reported that higher surfactant concentrations are required to reach a full molecular packing at the oil/water interface compared to the theoretically calculated values. Some studies also suggest the formation of multilayer islands of surfactants at the oil/water interface.^[32]^ The modified Poisson–Boltzmann model developed by Peng et al. suggests lower adsorption of SDS at the oil/water interface compared to that at the water/air interface.^[33]^ Shahir et al. argue that at the oil/water interface, the surfactant presence is 70 % lower than that at the water/air interface, as a consequence of stronger repulsion between the ionic SDS head groups at the oil/water interface.^[34]^ Similarly, Fainerman et al. emphasized that the competitive as well as cooperative adsorption between oil and surfactant molecules should be taken into account to provide a good description of the oil/water interface.^[35]^

During the surfactant‐induced ordering of MCT molecules, we assume a change in area per MCT molecule from 150 Å^2^ (low ordered) to ≈80 Å^2^ (fully ordered).^[36]^ Hence, surfactant molecules can fit in the gained space between ordered MCT molecules (≈3 surfactant per 1 MCT molecule). Indeed, the observed changes in the SFG spectra at these low surfactant concentrations reflect the high surface sensitivity of SFG spectroscopy compared to other analytical methods such as surface tension measurements.

The evolution of the OH‐signal intensity as a function of SDS coverage at the oil/water interface is plotted on the right axis of Figure 3, showing an increasing trend with increasing dSDS coverage. As was discussed earlier, the presence of charged head groups of dSDS at the buried oil/water interface is responsible for this increased OH‐signal intensity, confirming the co‐existence of oil, surfactant, and water molecules at the interface. Indeed, the large population of interfacial molecules (i.e., MCT oil molecules and dSDS chains) correlates with higher molecular ordering, consistent with other studies. For instance, increasing surfactant concentration was reported to increase the molecular ordering of SDS at the water/CCl_4_ interface.^[8]^ Other macromolecules such as proteins also show higher molecular packing and increased organization with increasing interfacial density.^[37]^

Once the population of interfacial molecules reaches its maximum, the further addition of dSDS in the bulk solution does not change the CH‐signal intensities anymore (See Figure 2 b). This observation can be explained by the fact that the interfacial molecules are already densely packed and no space is left for additional surfactant molecules to adsorb. Recent studies suggest that the excess surfactant molecules at the oil/water interface repel the oil molecules from the interface.^[35]^ Moreover, at high coverages, repulsive electrostatic forces from the ionic surfactant head can hinder the adsorption of further SDS molecules at the oil/water interface.

To correlate the molecular insights at the buried oil/water interface to macroscopic emulsion properties, we prepared MCT in water emulsions (as described in the SI). The emulsion stability was evaluated as a function of SDS coverage as schematically shown in Figure 3. With no or very low surfactant coverage, the oil phase could not be fully dispersed in the aqueous phase. At a sufficiently high surfactant coverage (corresponding to the full ordering of the oil molecule and increased electrostatic repulsion) well dispersed and stable emulsions could be obtained.

As mentioned earlier, the SFG spectra reported so far are acquired at equilibrium. Nevertheless, the kinetics of surfactant adsorption and the age of surfaces play an important role in system stability, for instance during emulsion preparation and for long term stability of emulsions. Many studies have been devoted to characterizing surfactant adsorption kinetics to oil/water interfaces. Here, we utilize SFG spectroscopy to assess the adsorption of surfactant to the buried oil/water interface over time and its consequent impact on the conformation and order of interfacial water and oil molecules.

**Figure 4:**
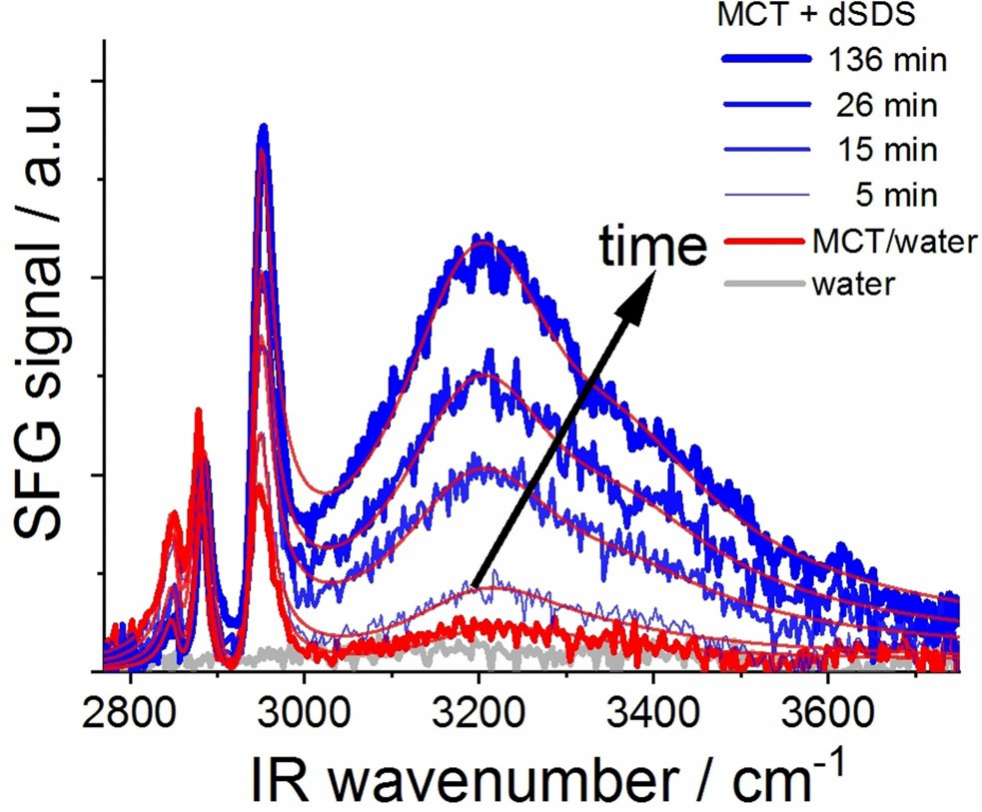
Time dependence SFG spectra at the buried MCT oil/water interface after addition of 31.9 μmol L^−1^ dSDS to the aqueous phase. The thin lines are the fits to the experimental data points.

An example of the time evolution of the SFG spectra for the dSDS bulk concentration of 31.9 μmol L^−1^ is provided in Figure 4. The noted time is the starting time of measurement after the addition of dSDS to the bulk water phase underneath the MCT oil/water interface. The MCT oil/water interface was already at its equilibrium before the addition of dSDS to the solution. At such relatively low surfactant concentrations (31.9 μmol L^−1^), dSDS molecules despite their surface affinity need time to reach their adsorption equilibrium at the oil/water interface. As can be seen in this Figure, after 5 minutes from the introduction of surfactant to the system, only a minor increase in the SFG signal at the OH‐region is observable, compared to that of the system in the absence of surfactant. At this time only a small fraction of bulk surfactant molecules adsorb to the buried oil/water interface, thus minimally aligning interfacial water molecules. The intensity of the OH‐signal increases with time until it reaches a maximum value (after approximately two hours) after which no further change in the SFG spectra could be detected. As reported elsewhere,^[38]^ the kinetics of the surfactant adsorption to the interface are also dependent on the bulk surfactant concentration. Therefore, we performed time‐dependent adsorption studies at two different surfactant concentrations (i.e., 31.9 μmol L^−1^ and 191.4 μmol L^−1^), as will be discussed later.

**Figure 5:**
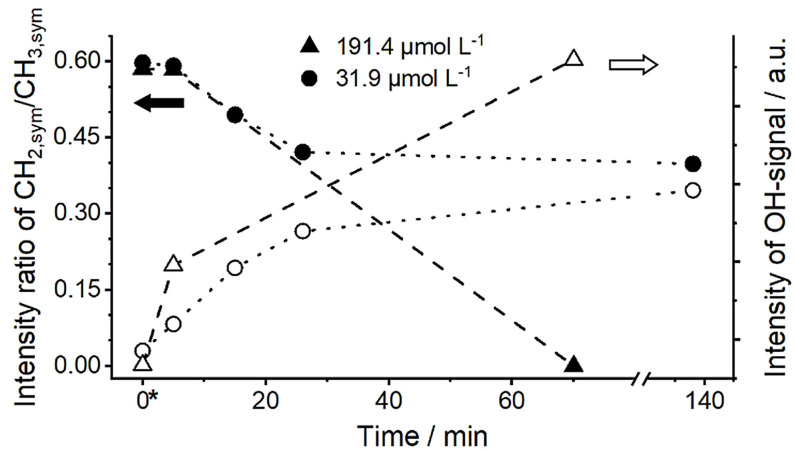
The ratio of the fitted peak amplitude of CH_2_/CH_3_ (filled symbols, left axis) and OH‐signal intensity (open symbols, right axis) over time for two different bulk dSDS concentrations at the buried MCT oil/water interface. Lines are to guide the eyes. The corresponding values in the absence of surfactant are displayed as time 0*. The estimated maximum average error was ±6 %. The right scale does not start from 0.

Gradual adsorption of surfactant from the bulk water phase to the oil/water interface (results in Figure 4 and Figure S4 in the SI) is accompanied by alterations in the CH‐region of the SFG spectra. In Figure 5, the fitted amplitude ratios of CH_2,sym_/CH_3,sym_ peaks from interfacial MCT oil molecules at the buried oil/water interface are reported for two bulk dSDS concentrations as a function of time. The right axis in this Figure shows the corresponding OH‐signal amplitudes as a measure for the amount of surface‐adsorbed ionic surfactant.

As can be seen in Figure 5, for both low and high bulk dSDS concentrations (i.e., 31.9 μmol L^−1^ and 191.4 μmol L^−1^) the fitted amplitude ratio of CH_2,sym_ to CH_3,sym_ decreases over time. Nevertheless, in the presence of low surfactant concentration, this ratio drops by a maximum of ≈60 % (up to 30 minutes, after which the ratio remains unchanged), whereas when higher dSDS concentration is added to the system, the CH_2,sym_ to CH_3,sym_ peak ratio drops more significantly and approaches zero after about 60 minutes (after which the spectra remain unchanged and the “full ordering” is reached). Comparing the results in Figures 3 and 5, it becomes evident that the conformation of interfacial oil molecules depends upon both bulk surfactant concentration and the age of the interface.

The increase in the OH‐signal intensity for the low and high bulk dSDS concentrations (right axis in Figure 5) is consistent with the above‐mentioned observations. The slope in the increased OH‐signal intensity is larger in the presence of the higher surfactant concentrations. With the bulk dSDS concentration of 31.9 μmol L^−1^, after the initial increase of the OH‐signal amplitude (until approximately 30 minutes) the signal intensity gradually increases whereas at the higher dSDS concentration (≈190 μmol L^−1^), the initial rise in the signal intensity is more significant and a much higher OH‐signal intensity is observed after approximately 60 minutes.

Within the time resolution of our SFG measurements (3–5 minutes), the decrease of the CH_2,sym_ to CH_3,sym_ peak intensity ratio (i.e., increased order of interfacial oil molecules) and the increased OH‐amplitude initially follow relatively similar slopes at both bulk surfactant concentrations. As was described earlier, both effects are directly connected to the adsorption of dSDS to the buried oil/water interface. Therefore, we conclude that the intercalation of the aliphatic chains (i.e., the hydrophobic tail of dSDS and hydrocarbon chains of MCT) happens shortly after the adsorption at the interface. Indeed, the molecular structure at the buried oil/water interface affects macroscopic properties of the interface such as interfacial tension. Therefore, we performed complementary surface tension measurements at the interface between MCT oil and bulk water phase in the presence of different surfactant concentrations in the aqueous phase.

**Figure 6:**
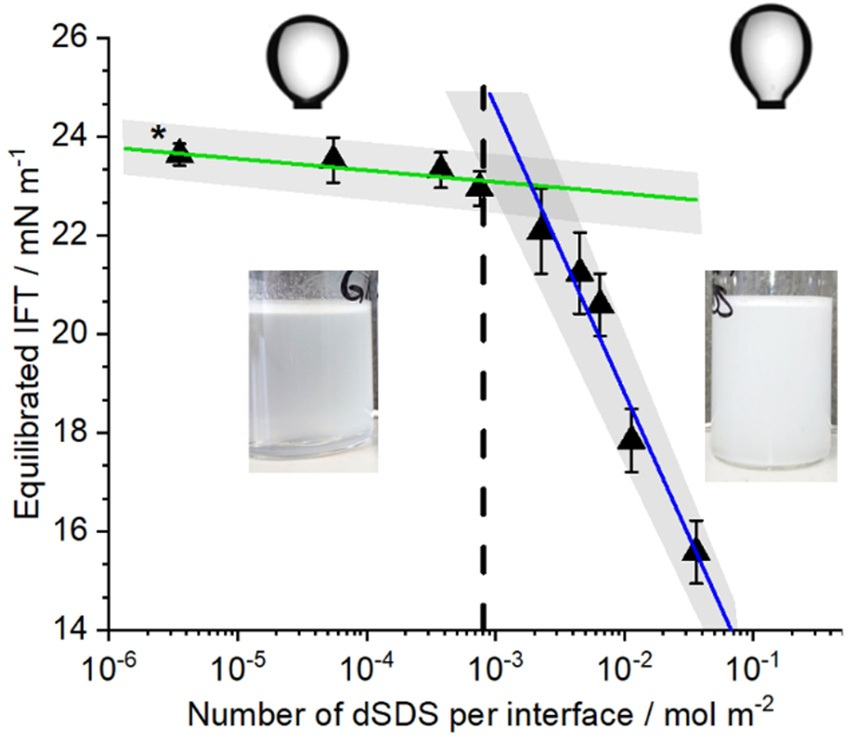
Changes of the interfacial tension between MCT oil and water with increasing surfactant concentration. The bulk concentration of surfactant is normalized to the available interfaces, as was discussed in the text. Linear fits to the experimental data points are shown as continuous lines. The vertical dashed line is for comparison with SFG experiments and denotes the corresponding bulk dSDS concentration that results in the “full ordering” of the alkyl chains of interfacial MCT oil molecules. * denotes the interfacial tension between MCT oil and water in the absence of surfactant. Insets are the shape of oil droplets (in surface tension measurement) and photographs of the prepared emulsions. With low SDS concentration an emulsion with low stability is formed (note the phase separation at the bottom of the container), whereas with high SDS concentration, the emulsion is macroscopically stable.

Surfactants are known, from experiments and simulations, to lower the IFT.^[39]^ In Figure 6, the change in the measured equilibrium IFT at the oil/water interface is presented as a function of surfactant concentrations, comparable to and beyond those used in our SFG measurements. The reported IFT values are at equilibrium and remained constant for several hours. The surfactant concentration is normalized to the total available interface to enable a direct comparison with the used surfactant concentrations of the SFG measurements. The oil/water interface in the SFG measurements is considerably larger than the interface of the oil droplet used during the pendant drop experiments. Moreover, surfactant molecules adsorb at the water/air interface of the cuvette as well. Therefore, the used dSDS concentration was normalized to the overall surface area of the droplet interface and the water/air interface of the cuvette.

As displayed in Figure 6 the IFT between oil and water decreases with increasing surfactant concentration, with two different slopes. At low interfacial dSDS concentrations (<7.5×10^−4^ mol m^−2^) the interfacial tension shows only a minor decrease compared to the IFT value of the oil/water interface in the absence of surfactant. With the further addition of dSDS, beyond this initial concentration, a significant decrease in the interfacial tension occurs. A similar trend was described by Menger et al.^[40]^ for SDS adsorption at the water/air interface, where a minimal change of IFT was detected with low surfactant concentrations. The authors observed a steep decrease of IFT values with increasing SDS concentration after surpassing 80 % of the maximum surfactant coverage. The two slopes in Figure 6 overlap at a transition zone, at the corresponding concentration (8.1×10^−4^ mol m^−2^) which is comparable to the dSDS concentration where the “full ordering” of the MCT oil alkyl chains is observed with SFG (see Figures 2 and 3). Based on these observations, the increased ordering of alkyl chains of the interfacial oil molecules at the buried oil/water interface, owing to the intercalation of hydrophobic tails of the surfactant, seems to be a prerequisite for a significant change of the IFT between the oil and aqueous phase. This conclusion is consistent with the observations by Müller et al. that SDS clustering reduces the interfacial tension at the oil/water interface by changing the organization of the oil molecules.^[29]^ Fainerman et al. also explained the change in interfacial tension at the oil/water interface with the increased ordering of both ionic surfactant and alkane molecules, far below the CMC value. They proposed a two‐step model that describes the adsorption of surfactants at the oil/water interface; Initial cooperative effects enhance the surfactant adsorption at the oil/water interface compared to the water/air interface, whereas, at higher surfactant concentrations, competitive adsorption between oil and surfactant molecules leads to a depletion of the oil molecules from the interface.^[35]^

Comparing the results from SFG measurements and IFT measurements, it becomes evident that the presence and interfacial concentration of surfactants, the age of the interface, as well as the rearrangement of the interfacial molecules (oil, water, and surfactant) are all effective parameters altering the molecular and macroscopic properties of the oil/water systems, for instance in emulsions. Our results in this study show that SFG spectroscopy at the buried oil/water interface not only unravels the molecular interactions between oil, water, and surfactant molecules, as well as their corresponding conformational changes at a molecular level, but also that the obtained fundamental information can describe the macroscopic physicochemical properties of the oil/water interface such as surface tension. Such a fundamental understanding allows the systematic formulation of stable emulsions. Nevertheless, the impact of the surface curvature in emulsion systems containing small droplets (i.e., in the order of hundred nm in diameter) on the oil/water interfacial properties and the observed differences with planar oil/water interface should not be overlooked. As was described by Smolentsev et al.^[41]^ using SFS, some interfacial properties such as the strength of hydrogen bonding differ between the planar oil/water systems and systems containing small droplets.

## Conclusion

Herein, we utilized SFG spectroscopy to unravel molecular interactions at the buried MCT oil/water interface in the presence of anionic surfactant (SDS) at different concentrations. The systematic combination of protonated and deuterated oil and surfactant, as was performed in this research, at the oil/water interface allowed the spectroscopic examination of both surfactant and oil molecules at the buried oil/water interface. Our results indicated that the adsorption of surfactants to the buried oil/water interface increases the electric field at the interface and simultaneously increases the degree of ordering of the interfacial oil due to the intercalation of aliphatic chains of surfactant and oil molecules. The impact of molecular conformation and magnitude of the interfacial electric field was connected to macroscopic emulsion stability. At sufficiently high surfactant concentrations, the *gauche* defects in the alkyl chains of the interfacial oil molecules are completely eliminated and MCT oil molecules arrange in an *all trans* conformation, all of which result in well dispersed oil‐in‐water droplets and a stable emulsion.

The chemical structure of the oil, the impact of surfactant concentration and the age of the formed interface were connected to the macroscopic IFT between water and oil. While the low SDS surface coverage only minimally affected the IFT, a significant reduction in IFT was observed at SDS surface coverages which correspond to the “full ordering” of the interfacial oil molecules. These results obtained from the buried oil/water interface are the first experimental spectroscopic evidence on the reciprocal impact of oil and surfactant molecules on the molecular interface structure and its macroscopic impact on emulsion properties.

## Conflict of interest

The authors declare no conflict of interest.

## Supporting information

As a service to our authors and readers, this journal provides supporting information supplied by the authors. Such materials are peer reviewed and may be re‐organized for online delivery, but are not copy‐edited or typeset. Technical support issues arising from supporting information (other than missing files) should be addressed to the authors.

Supporting Information
