# Supplementary material for: Effect of Surfactants on the Molecular Structure of the Buried Oil/Water Interface
Source: Angew Chem Int Ed Engl. 2021 Sep 14;60(47):25143–50. doi: 10.1002/anie.202110091 (PMC9293143; doi:10.1002/anie.202110091)
Supplement: Supplementary file 1 — Supporting Information [file ANIE-60-25143-s001.pdf]

Angewandte Chemie
Eine Zeitschrift der Gesellschaft Deutscher Chemiker

Supporting Information

Effect of Surfactants on the Molecular Structure of the Buried Oil/Water Interface

Saman Hosseinpour[+], Vanessa Götz[+], and Wolfgang Peukert**

anie_202110091_sm_miscellaneous_information.pdf

Table of Contents

1. Materials and Methods
1.1. Materials
1.2. Methods
1.2.1. SFG
1.2.2. Emulsion Preparation
1.2.3. Interfacial Tension
2. Fitting the SFG Spectra
3. Additional SFG Spectra
4. Supporting References

WILEY-VCH

1. Materials and Methods

1.1. Materials

SDS (Sigma Aldrich), deuterated sodium dodecyl-d25 sulfate (dSDS, >= 98 atom %, Sigma Aldrich), MCT (approximately 60% octanoic acid, 40 % decanoic acid, WITARIX®, IOI Olea GmbH), and acetone (>=99.9%, GC Ultra Grade, Carl Roth) were used as received. All glassware was cleaned using Alconox® precision cleaner and submerged in a sulfuric acid bath (98%, p.a., Carl Roth) containing approximately 20 g/l Nochromix® (Sigma-Aldrich), for a minimum of 12 hours before use. The glassware was rinsed with a copious amount of ultrapure water (Milli-Q®, 18.2 MΩ cm) and used immediately afterward. Unless explicitly mentioned, the ionic strength of the solutions is limited to the ionization of dSDS in the bulk solution.

1.2. Methods

1.2.1. SFG

Understanding the molecular interactions like van der Waals (vdW) attraction, steric hindering, charge induced repulsive forces, or competitive adsorption at the interfaces between water, surfactant, and oil phases in an emulsion system is crucial for the formulation and optimization of stable emulsion systems.[1] Since the number of interface molecules is several orders of magnitudes smaller than that in the bulk phases, investigating the molecular characteristics at interfaces is experimentally challenging.
Several analytical methods, such as static and dynamic surface tension measurements,[2] fluorescence based techniques,[3] X-ray scattering,[4] small angle neutron scattering (SANS), and neutron reflectivity[5] have been used so far to investigate the adsorption behavior of surfactant molecules at the interface between two immiscible liquid phases. Nevertheless, these methods either require exhaustive sample preparation and labeling, need expensive high-energy sources, or have limited ability to provide molecular information on surfactants at the buried oil/water interface. Therefore, in this study we utilized inherently surface sensitive SFG to unravel the molecular interactions at the buried water/oil interface in the presence of adsorbing surfactants.
The details of the experimental SFG setup that is used in this study are provided elsewhere.[6] Briefly, SFG measurements were performed by overlapping an etalon filtered visible (vis) pulse (ω_{vis}=800 nm, full-width half-maximum ($FWHM_{vis}$)= ~18 cm^{-1}) and a tunable infrared beam ($FWHM_{IR}$= 250 cm^{-1}, tuned from 2755 cm^{-1} to 3585 cm^{-1}) in time and space at the liquid/air interface. The angle of the incident for the vis and IR beams was 54° and 60° to the surface normal, respectively. The reflected vis and IR beams from the sample surface were optically and spatially filtered out and the generated SFG signal was directed toward a spectrograph (Shamrock 303i) and was recorded using a charge-coupled device (CCD Andor, iStar). The polarization of SFG, vis, and IR beams was set to s, s, and p (hereafter referred to as ssp polarization combination).
For the SFG measurements, first MCT was dissolved in acetone (0.05 wt% MCT). Then, 10 µl of the dissolved MCT was drop casted on top of the water phase. This sample deposition procedure enabled the spreading of a controlled and thin film oil layer at the surface of the water. Based on the added volume of the MCT (initially dissolved in acetone and drop cast on the surface) we estimated the average MCT layer thickness of <10 nm. Using controlled SFG measurements (not shown here), it was ensured that the added acetone was evaporated before the measurements at the oil/water interface. SDS and MCT oil both contain hydrocarbons and thus both contribute to SFG signal generation in the CH stretching region. To distinguish the individual contribution of surfactant and oil in the overall SFG signal, deuterated SDS (dSDS) was used, as will be discussed later. A stock solution of dissolved dSDS in Milli-Q water (32 mmol/l) was prepared and injected into the water phase (total volume of 10 ml) in an appropriate volume to reach the desired concentration of surfactant in the bulk solution. If needed, the amount of dSDS was gradually increased in the bulk water phase. Nevertheless, the maximum bulk concentration of SDS in this study was far below the CMC value for SDS (7.9 mmol/l at 25°C).[7]
The liquid samples were placed in a glass container (5.5 cm inner diameter) which was rotated during the SFG measurement, to avoid thermal displacement of the interfacial molecules by laser beams.[8] The rotation speed of the sample container was set to avoid any agitation of the liquid surface. All the acquired SFG spectra were normalized against the nonresonant SFG signal of a polycrystalline gold film that was cleaned with air plasma. All SFG spectra were acquired at room temperature and with the exception of the kinetic measurements, all were taken after the samples reached an equilibrium state. SFG spectra of sufficient signal-to-noise ratio could be obtained with approximately 5 minutes of signal acquisition, which sets the time resolution limit for kinetic measurements.

1.2.2. Emulsion Preparation

To evaluate the macroscopic emulsion stability, oil in water emulsions stabilized by SDS were prepared as follows: 1 vol% MCT was added into SDS solutions (45 ml) of different concentrations. Oil and surfactant solution was mixed by dispensing 5 ml of the sample five times using a pipette to form a pre-dispersed emulsion of low stability. The final emulsification was carried out with the aid of a T18

WILEY-VCH

Ultra-Turrax® equipped with a S18N-10G dispersion tool (IKA) with 11200 rpm for 1.5 min. followed by 7200 rpm for 3.5 min. We defined a stable emulsion when the whole oil phase could be dispersed and no phase separation occurred within 1 hour.

1.2.3. Interfacial Tension

A drop shape analyzer DSA100 (Krüss) in the invert pendant drop mode was used to determine the interfacial tensions between water and MCT oil, in the presence of different concentrations of SDS in the bulk aqueous phase. Since MCT oil has a lower density than water, using the controlled pump in the device, a defined volume of oil was pushed out of a syringe into an upward bent steel needle inside a squared cuvette filled with Milli-Q water (total volume of 30 ml). The cuvette was put in the acid bath and was cleaned thoroughly between measurements to ensure the removal of surfactant and oil residues. The shape of the oil droplet was recorded over time and the Young- Laplace equation was fitted to the recorded droplet shapes to acquire the interfacial tension values. After reaching an equilibrium interfacial tension between MCT oil droplet and water phase (i.e., no further change of interfacial tension over time), the SDS from a stock solution was gradually added to the bulk water phase in the cuvette to evaluate the effect of SDS on surface tension between water and MCT oil. A cover was put on the top of the cuvette to prevent water evaporation during surface tension measurements to minimize the possible errors. The interfacial tension measurements were performed at room temperature (with ±1ºC temperature fluctuations).

2. Fitting the SFG Spectra

Under dipole approximation, SFG spectra provide molecular information of the species residing in a non-centrosymmetric environment, for instance, at surfaces and interfaces with broken molecular symmetry. As described in Equation 1, the SFG signal intensity (I_{SFG}) is proportional to the intensity of the fundamental beams (I_{vis} and I_{IR}) and the square sum of the non-resonant and resonant second-order electric susceptibility ($\chi_{NR}^{(2)}$ and $\chi_{R}^{(2)}$, respectively), both of which are complex entities. The $\chi_{NR}^{(2)}$ is a frequency independent term and the total I_{SFG} will be resonantly enhanced when the frequency of the IR beam (ω_{IR}) coincides with the frequency of nth vibrational mode (ω_n):

$$\mathrm{I_{SFG}} \propto \left|\chi_{NR}^{(2)} e^{i\gamma} + \sum_n \frac{A_n}{\omega_n - \omega_{IR} + i\Gamma_n}\right|^2 . I_{vis} . I_{IR} \qquad (1)$$

where A_n and Γ_n denote the amplitude and the damping constant of nth vibrational mode, respectively and γ is described as the phase difference between the $\chi_{NR}^{(2)}$ and $\chi_{R}^{(2)}$.

$\chi_{R}^{(2)}$ related to molecular hyperpolarizability (β), the number of contributing oscillators (N), and the dielectric permittivity (ε_0), according to Equation (2);

$$\chi_{R}^{(2)} = \frac{N}{\varepsilon_0} \langle \beta_R^{(2)} \rangle \qquad (2)$$

where the brackets indicate an average over all molecular orientations. Therefore, an increased number of interfacial molecules and their ordered structure result in enhanced SFG signal intensity.[9]

At the electrified surfaces, the interfacial potential ($\Phi(0)$) interacts with both $\chi^{(2)}$ and third-order susceptibility ($\chi^{(3)}$). Hence, equation (1) for electrified surfaces can be extended to:

$$\mathrm{I_{SFG}} \propto \left|\chi_{NR}^{(2)} e^{i\gamma} + \sum_n \frac{A_n}{\omega_n - \omega_{IR} + i\Gamma_n} + \chi^{(3)} \frac{\kappa}{\sqrt{\kappa^2 + (\Delta k)^2}} e^{i\varphi} \Phi(0)\right|^2 \qquad (3)$$

in which κ and Δk are the inverse Debye screening length and inverse coherence length of the SFG process, respectively. In equation (3), φ denotes the phase-matching factor, and is defined as:[10]

$$\varphi = arctan\left(\frac{\Delta k}{\kappa}\right) \qquad (4)$$

In Figure S 1, the calculated φ and corresponding Debye length are provided as a function of ionic strength.

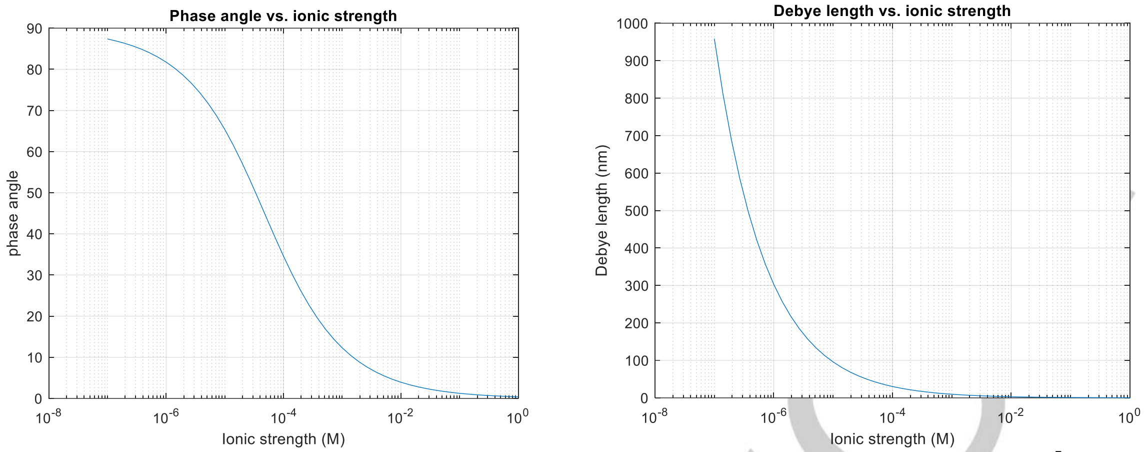

Figure S 1: The changes of the phase angle (left) and Debye length (right) as a function of ionic strength. The lowest ionic strength of 10^{-7} is based on the water autoionization.

As will be discussed in the following, the most significant impact of the $\chi^{(3)}$ for the buried oil/water interface in the presence of dSDS is on the OH-spectral region (i.e., from 3100-3700 cm^{-1}). Nevertheless, in the present work, we focus mainly on the relative changes of the CH_2 and CH_3 amplitudes (at the buried MCT oil/water interface as a function of bulk dSDS concentration), which reflect the increased ordering of the interfacial oil molecules as the dSDS concentration increases. This phenomenon occurs as a result of the intercalation of the hydrophobic tail of the dSDS within the interfacial MCT oil molecules and is minimally affected by the $\chi^{(3)}$ contribution. Hence, in our SFG spectral analysis, we make no distinction between the relative contribution of both $\chi^{(2)}$ and $\chi^{(3)}$ and used equation (1) for fitting our SFG spectra (using effective $\chi^{(2)}$, as was utilized by Das et al).[11]

The following spectral simulations (Figure S 2) show that, although at the sufficiently high electric field at the oil/water interface (at high dSDS concentrations), the magnitude of the SFG signal at the CH stretching region (i.e., between 2840-2960 cm^{-1}) is slightly affected by the $\chi^{(3)}$ contributions, the change of the relative ratio of the CH_2/CH_3 is negligible to non-existing. In the example, the surface potential is varied (-50 mV, -100 mV, -200 mV, -300 mV). The blue curve is the simulated SFG spectrum of the MCT/oil interface in the presence of the 2.3 µmol/l SDS. It should be noted that based on the Zeta potential measurements on the MCT/SDS/Water emulsion system with comparable surfactant concentration, the surface potential of around -100 mV was obtained.

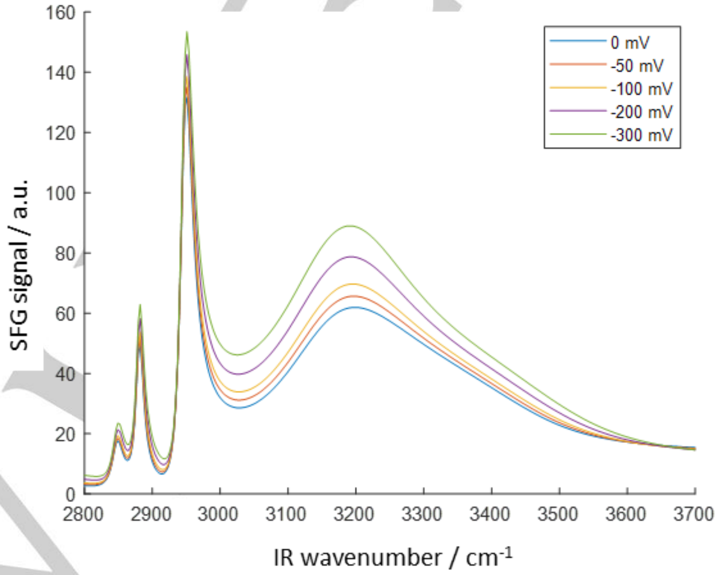

Figure S 2: Simulated SFG spectra as a function of interfacial potential, in which the interfacial potential interacts with both $\chi^{(2)}$ and $\chi^{(3)}$

As can be seen here, even with accounting for the three times higher surface potential (than the expected value based on Zeta potential measurement) the CH_2/CH_3 ratio is not greatly affected, whereas our experimental SFG results (see Figure 2 in the manuscript) clearly show that at high dSDS concentrations (>~190 µmol/l) the CH_2/CH_3 ratio approaches zero.
To further confirm that the determined CH_2/CH_3 ratio is not affected by the change of the Debye length, we also performed additional control measurements in which the ionic strength of the solution is increased by the addition of NaCl to the solution, e.g., from ~30 µM (for pure MCT-surfactant system) to 40 mM (for MCT-surfactant-NaCl). By adding 40 mM NaCl to a 32 µM dSDS-containing solution

(Figure S 3), the Debye lengths changes (~1 nm vs. ~50 nm, respectively). Also, the NaCl screens the interfacial charge at the buried oil/water interface (also known as double layer quenching). Assuming the initial surface potential of -200 mV for the low ionic strength system, the reduction of the SFG signal after the addition of the 40 mM NaCl can well be reproduced by accounting for both $\chi^{(2)}$ and $\chi^{(3)}$. Such control measurements were performed for all SDS concentrations and no change in the CH_2/CH_3 intensity ratios was detectable with high and low ionic strength whereas the reduction of the signal at the OH stretching region was clear.

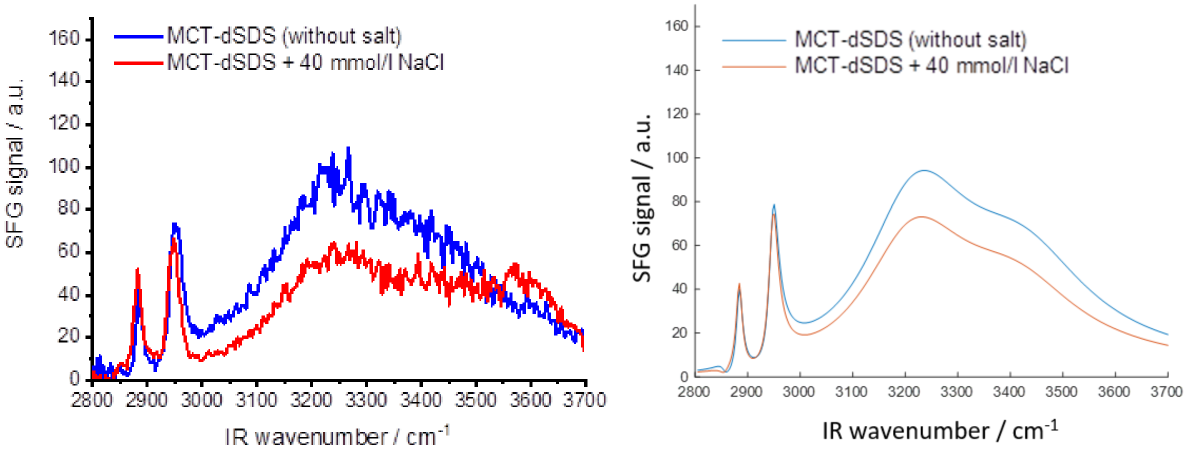

Figure S 3: Left panel) SFG spectra of the buried MCT oil/water interface in the presence of 32 µmol/l dSDS in the bulk solution without salt (blue spectrum) and with the addition of 40 mmol/l NaCl (red spectrum). Right panel) The fitted spectrum of MCT oil/water interface in the presence of 32 µmol/l dSDS in the bulk solution (blue) and with including 40 mmol/l ionic strength.

In the course of fitting, the transition frequency of the resonant peaks in the frequency range from 2750 - 3800 cm^{-1}, their linewidths, as well as the phase of non-resonant part of the signal were kept constant (see Table S1). The amplitudes of the resonant peaks were iteratively optimized to obtain the best fitting results.

Table S1. Parameters used for the fitting of SFG spectra. All spectra are fitted with -2pi (with 95% confidence interval) as the phase difference between the resonant and nonresonant signals. The amplitude signs (for individual vibrations) are in accordance with Mondal et al.[12]

Assignment	$CH_{2,sym}$	$CH_{3,sym}$	$CH_{3,FR}$	Strongly H-bonded water	Weakly H-bonded water
Frequency / cm^{-1}	2850	2875	2945	3194	3352
Widths	10	10	12	121	111
Fitted amplitudes (Figure 2)					
MCT	35.05	69.49	105.55	-569.07	0.00
0.2 µmol/l	32.28	66.19	104.44	-666.96	0.00
2.3 µmol/l	28.14	60.05	125.12	-1085.00	-160.92
31.9 µmol/l	31.90	80.08	136.05	-1212.67	-247.40
191.4 µmol/l	6.38E-05	79.57	120.90	-1896.85	-402.18
287.1 µmol/l	4.80E-07	68.76	121.81	-1829.89	-554.84
Fitted amplitudes (Figures 4 & 5 - 31.9 µmol/l)					
0 min	44.59	74.60	90.48	-429.57	0.00
5 min	41.70	70.48	99.65	-579.68	-22.22
15 min	36.04	72.80	120.56	-831.04	-131.06
26 min	28.63	67.90	131.80	-997.34	-200.50
136 min	31.90	80.08	136.05	-1212.67	-247.40
Fitted amplitudes (Figure 5 – 191.4 µmol/l)					

0 min	45.65	77.92	83.97	-338.51	6.38E-05
5 min	43.54	74.63	125.31	-866.39	-112.97
70 min	0.00	79.57	120.90	-1896.85	-402.18

3. Additional SFG Spectra

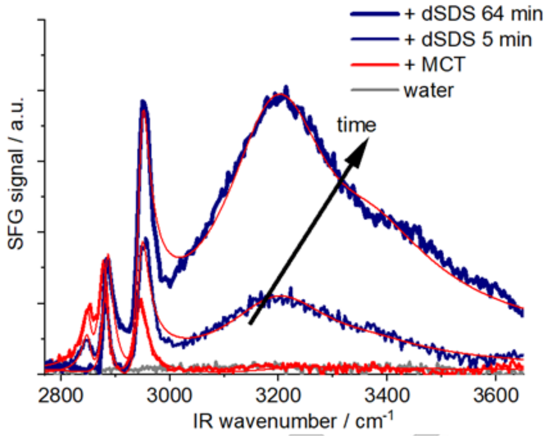

Figure S 4. Time dependence SFG spectra at the buried MCT oil/water interface after addition of 191.4 µmol/l dSDS to the aqueous phase

4. Supporting References

[1] M. J. Rosen, J. T. Kunjappu, *Surfactants and interfacial phenomena*, Wiley, Hoboken, N.J., **2012**.
[2] a) N. Mucic, N. M. Kovalchuk, V. Pradines, A. Javadi, E. V. Aksenenko, J. Krägel, R. Miller, *Colloids Surf., A* **2014**, *441*, 825; b) Z.-Y. Liu, Z.-Q. Li, X.-W. Song, J.-C. Zhang, L. Zhang, L. Zhang, S. Zhao, *Fuel* **2014**, *135*, 91; c) M. Mottola, B. Caruso, M. A. Perillo, *Sci. Rep.* **2019**, *9*, 2259.
[3] C. V. H.-H. Chen, Y. Liu, H. A. Stone, R. K. Prud'homme, *Langmuir* **2018**, *34*, 10512.
[4] M. L. Schlossman, A. M. Tikhonov, *Annu. Rev. Phys. Chem.* **2008**, *59*, 153.
[5] J. Penfold, R. K. Thomas, P. X. Li, J. T. Petkov, I. Tucker, J. R. P. Webster, A. E. Terry, *Langmuir* **2015**, *31*, 3003.
[6] a) C. Meltzer, J. Paul, H. Dietrich, C. M. Jäger, T. Clark, D. Zahn, B. Braunschweig, W. Peukert, *J. Am. Chem. Soc.* **2014**, *136*, 10718; b) K. Engelhardt, W. Peukert, B. Braunschweig, *Current Opinion in Colloid & Interface Science* **2014**, *19*, 207.
[7] F. Warsi, M. R. Islam, M. S. Alam, M. Ali, *Journal of Molecular Liquids* **2020**, *310*, 113132.
[8] E. H. G. Backus, D. Bonn, S. Cantin, S. Roke, M. Bonn, *J. Phys. Chem. B* **2012**, *116*, 2703.
[9] a) A. G. Lambert, P. B. Davies, D. J. Neivandt, *Appl. Spectrosc. Rev.* **2005**, *40*, 103; b) Y. R. Shen, *Fundamentals of Sum-Frequency Spectroscopy*, Cambridge University Press, Cambridge, **2016**.
[10] P. E. Ohno, H. Wang, F. M. Geiger, *Nat Commun* **2017**, *8*, 1032.
[11] S. Das, M. Bonn, E. H. G. Backus, *J. Chem. Phys.* **2019**, *150*, 44706.
[12] J. A. Mondal, S. Nihonyanagi, S. Yamaguchi, T. Tahara, *J. Am. Chem. Soc.* **2010**, *132*, 10656.
